# Disseminated *Mycobacterium abscessus* Infection and Showerheads, Taiwan

**DOI:** 10.3201/eid1711.110050

**Published:** 2011-11

**Authors:** Yu-Min Kuo, Aristine Cheng, Po-Chang Wu, Song-Chou Hsieh, Szu-Min Hsieh, Po-Ren Hsueh, Chia-Li Yu

**Affiliations:** National Taiwan University Hospital, Taipei, Taiwan

**Keywords:** Mycobacterium abscessus, bacteremic lymphadenitis, Sjögren syndrome, showerheads, bacteria, Taiwan, letter

**To the Editor:** Diseases caused by nontuberculous mycobacteria (NTM) in patients with Sjögren syndrome have rarely been reported (*1*,*2*). In addition, showerheads in residential bathrooms as a source of *Mycobacterium abscessus*–induced disseminated disease have never been reported (*3*–*5*).

A 65-year-old woman with Sjögren syndrome sought treatment at National University Taiwan hospital with fever (38.6°C) and a 3-month history of lymphadenopathy over the left neck, left submandibular, and bilateral inguinal areas. Active Sjögren syndrome with lymphadenitis was considered because of progressive hypergammaglobulinemia (IgG 3,030 mg/dL, reference range 700–1,600 mg/dL) and high titers of anti–Sjögren syndrome (SS) A (561 AU/mL) and anti-SSB antibodies (220 AU/mL; positive >120 AU/mL). A chest radiograph obtained 1 month before admission showed no active lung lesions; however, cultures of 3 samples of sputum all yielded *M. abscessus* bacteria (isolate A). Pathologic examination of excised lymph nodes of the bilateral inguinal area showed reactive lymphoid proliferation and granulomatous inflammation with multinucleate giant cell formation, suggestive of mycobacterial disease; however, there was no evidence of caseating necrosis or acid-fast bacilli. ELISA results were negative for antibodies to HIV-1, HIV-2, HTLV-1, and HTLV-2.

Parenteral antimicrobial drugs (imipenem, 500 mg every 8 h) and amikacin (250 mg 2×/d) along with oral clarithromycin (500 mg 2×/d) were administered. Fever subsided 3 days after lymph node excision with concomitant administration of antimycobacterial agents. The patient was treated successfully with intravenous antimicrobial drugs for a total of 14 days, followed by oral clarithromycin (500 mg 2×/d) and doxycycline (100 mg 2×/d) therapy for 4 months. Follow-up blood cultures 10 weeks after initiation of antimycobacterial agents were negative for the organism. *M. abscessus* bacteria grew on cultures of the excised lymph nodes (isolate B) and 2 sets of blood cultures (isolate C)*.*

A total of 6 swab specimens taken from the interior surface of the showerheads from the 6 bathrooms of the patient’s 2 houses (3 in each house), 1 in Taichung (central Taiwan) and the other in Taipei (northern Taiwan), and 6 shower water samples of the 6 bathrooms were submitted for mycobacterial cultures. Four of the 6 swab samples (isolates D–G), 2 (isolates D and E) from Taichung and 2 (isolates F and G) from Taipei, grew *M. abscessus* bacteria. Cultures of shower water from the 6 bathrooms were all negative for the organism. These isolates were identified as *M. abscessus* by conventional biochemical methods and confirmed by 16S rRNA gene sequencing analysis and a PCR–restriction fragment length polymorphism–based method targeting a 439-bp fragment of the 65-kDa HSP gene as previously described (*6*,*7*). Random amplified polymorphic DNA patterns of these isolates (isolates A–G) as determined by means of arbitrarily primed PCR using 3 different random primers were identical (i.e., they shared every band) (Figure). Three unrelated isolates of *M. abscessus* recovered from cutaneous lesions of 3 patients who were treated at the same hospital in 2010 had distinct random amplified patterns that differed from those generated from isolates A–G (Figure).

**Figure:**
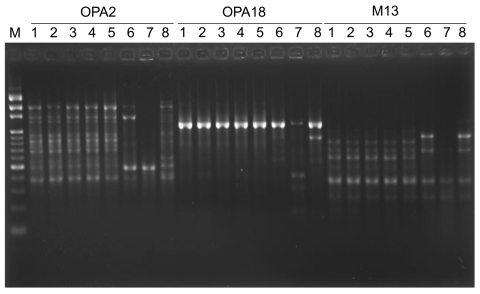
Random amplified polymorphic DNA patterns of 8 isolates of *Mycobacterium abscessus* generated by arbitrarily primed PCR with the primers OPA2, OPA18, and M13 (Operon Technologies, Inc., Alameda, CA, USA). Lanes: M, molecular size marker (1-kb ladder; Gibco BRL, Gaithersburg,MD, USA); 1, isolate A; 2, isolate B; 3, isolate C; 4, isolate D; 5, isolate F; 6–8, three unrelated isolates of *M. abscessus* recovered from cutaneous lesions of 3 patients who were treated at National Taiwan University hospital in 2010 (see text for designation of isolates).

A previous study in Taiwan showed that the incidence (no. cases/100,000 inpatients and outpatients) of all pulmonary disease caused by NTM increased significantly from 2.7 (1.26) in 2000 to 10.2 (7.94) in 2008 (*6*). The most common organism in localized pulmonary infection and disseminated infection was *Mycobacteriam avium* cellular complex, and *M. abscessus* predominated in skin and soft tissue infection and lymphadenitis (*6*,*8*). The rise in pulmonary infections or colonization by NTM over recent decades, particularly among immunocompromised populations, is reported to be partly associated with the increased use of showers (*3*–*5*,*9*). Recently, a few studies have shown a link between pulmonary *M. avium* complex infections and home showerhead water microbiology (*3*,*4*). Although pulmonary disease caused by *M. abscessus* did not develop in the patient reported here, multiple respiratory specimens showed evidence of pulmonary colonization. The fact that cultures of the swabs taken from the interior surface of 4 showerheads were positive for *M. abscessus* but that cultures of the shower water were negative for the organism support previous findings that assemblages of NTM can occur inside biofilm that forms on the interior surface of showerheads (*5*). The same strains of *M. abscessus* isolated from different showerheads suggested the possibility of contamination in the environment by the aerosolized microorganism from respiratory secretions of the patient.

The mechanisms of susceptibility to mycobacterial infection in the patient with Sjögren’s syndrome remain unknown (*1*,*2*). Previous studies suggest that toll-like receptor 2, dectin-1, tumor necrosis factor–α, interferon-γ, leptin, T-cells, and possibly neutrophils are major components in the host defense of HIV-noninfected patients against rapidly growing mycobacterial infections, including those caused by *M. abscessus* (*10*).

In summary, we report a case of bacteremic lymphadenitis caused by *M. abscessus* in a patient with Sjögren syndrome. Our data provide evidence that the interior surface of showerheads may serve as a source of infection by this waterborne and aerosolized microorganism.

## References

[1] Uji M, Matsushita H, Watanabe T, Suzumura T, Yamada M. A case of primary Sjögren's syndrome presenting with middle lobe syndrome complicated by nontuberculous mycobacteriosis [in Japanese]. Nihon Kokyuki Gakkai Zasshi. 2008;46:55–9. 18260312

[2] Tan HH, Tan A, Theng C, Ng SK. Cutaneous *Mycobacterium haemophilum* infections in immunocompromised patients in a dermatology clinic in Singapore. Ann Acad Med Singapore. 2004;33:532–6. 15329772

[3] Nishiuchi Y, Maekura R, Kitada S, Tamaru A, Taguri T, Kira Y, The recovery of *Mycobacterium avium*–intracellulare complex (MAC) from the residential bathrooms of patients with pulmonary MAC. Clin Infect Dis. 2007;45:347–51. 10.1086/519383 17599313

[4] Falkinham JO, Iseman MD, Haas P, Soolingen D. *Mycobacterium avium* in a shower linked to pulmonary disease. J Water Health. 2008;6:209–13. 18209283 10.2166/wh.2008.032

[5] Feazel LM, Baumgartner LK, Peterson KL, Frank DN, Harris JK, Pace NR. Opportunistic pathogens enriched in showerhead biofilms. Proc Natl Acad Sci U S A. 2009;106:16393–9. 10.1073/pnas.0908446106 19805310 PMC2752528

[6] Lai CC, Tan CK, Chou CH, Hsu HL, Liao CH, Huang YT, Increasing incidence of nontuberculous mycobacteria, Taiwan, 2000–2008. Emerg Infect Dis. 2010;16:294–6. 20113563 10.3201/eid1602.090675 PMC2958002

[7] Yakrus MA, Hernandez SM, Floyd MM, Sikes D, Butler WR, Metchock B. Comparison of methods for identification of *Mycobacterium abscessus* and *M. chelonae* isolates. J Clin Microbiol. 2001;39:4103–10. 10.1128/JCM.39.11.4103-4110.2001 11682537 PMC88494

[8] Chou CH, Chen HY, Chen CY, Huang CT, Lai CC, Hsueh PR. Clinical features and outcomes of disseminated infections caused by nontuberculous mycobacteria in a university hospital in Taiwan, 2004–2008. Scand J Infect Dis. 2011;43:8–14. 10.3109/00365548.2010.519345 20849364

[9] van Ingen J, Blaak H, de Beer J, de Roda Husman AM, van Soolingen D. Rapidly growing nontuberculous mycobacteria cultured from home tap and shower water. Appl Environ Microbiol. 2010;76:6017–9. 10.1128/AEM.00843-10 20639378 PMC2935072

[10] Chan ED, Bai X, Kartalija M, Orme IM, Ordway DJ. Host immune response to rapidly growing mycobacteria, an emerging cause of chronic lung disease. Am J Respir Cell Mol Biol. 2010;43:387–93. 10.1165/rcmb.2009-0276TR 20081053

